# Generalized Lymphadenopathy: Unusual Presentation of Prostate Adenocarcinoma

**DOI:** 10.1155/2011/439732

**Published:** 2011-08-23

**Authors:** Bulent Cetin, Zeynep Cetin, Suleyman Buyukberber, Ipek Isık Gonul, Ilgin Sahiner, Ugur Coskun, Mustafa Benekli

**Affiliations:** ^1^Division of Medical Oncology, Department of Internal Medicine, Faculty of Medicine, Gazi University, Ankara, Turkey; ^2^Department of Medical Oncology, Faculty of Medicine, Gazi University, Besevler, 06500 Ankara, Turkey; ^3^Department of Internal Medicine, Faculty of Medicine, Gazi University, Ankara, Turkey; ^4^Department of Pathology, Faculty of Medicine, Gazi University, Ankara, Turkey; ^5^Department of Nuclear Medicine, Faculty of Medicine, Gazi University, Ankara, Turkey

## Abstract

Generalized lymphadenopathy is a rare manifestation of metastatic prostate cancer. Here, we report the case of a 59-year-old male patient with supraclavicular, mediastinal, hilar, and retroperitoneal and inguinal lymphadenopathy, which suggested the diagnosis of lymphoma. There were no urinary symptoms. A biopsy of the inguinal lymph node was compatible with adenocarcinoma, whose prostatic origin was shown by immunohistochemical staining with PSA. The origin of the primary tumor was confirmed by directed prostate biopsy. We emphasize that a suspicion of prostate cancer in men with adenocarcinoma of undetermined origin is important for an adequate diagnostic and therapeutic approach.

## 1. Introduction

 Prostate cancer is the most common malignancy and the second most common cause of cancer-related death in men in the United States. The prognosis of prostate cancer is mainly determined by the presence or absence of metastases [1]. Most of the metastases occur in the bone, the other sites are lung, liver, pleura, and adrenals [2]. Lymphatic metastasis is rare. So, we present below a prostate cancer with common lymph node and metastases without symptoms initially. We present herein a case with rare manifestation of prostatic carcinoma initially mimicking malignant lymphoma due to supraclavicular lymphadenopathy and a paraaortic lymph nodes on F-18-fluorodeoxyglucose-(FDG-) positron emission tomography (PET/CT).

## 2. Case Report

**Figure 1:**
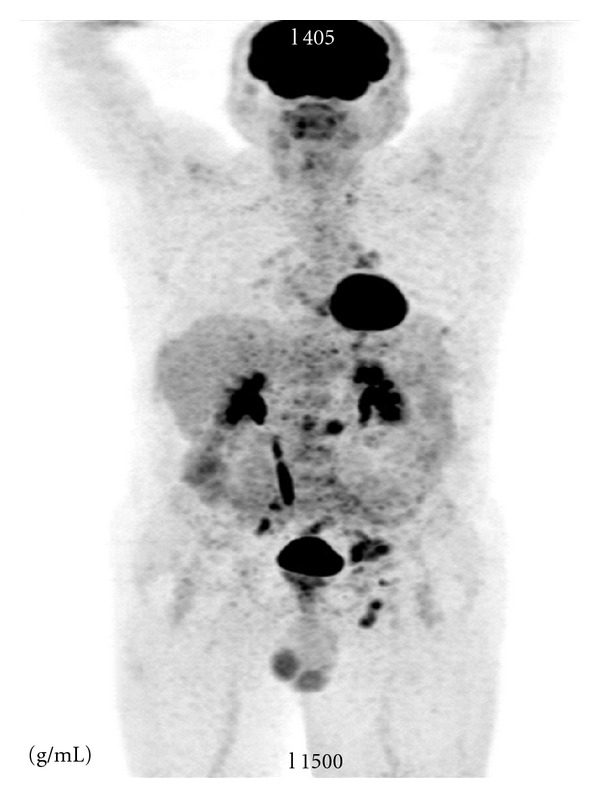
PET CT showed pathological increased FDG involvement of the left supraclavicular (suvmax: 4.5), aorticopulmonary (suvmax: 3.6), paratracheal (suvmax: 4.1), left tracheobronchial (suvmax: 5), left hilar (suvmax: 4.95), superior phrenic (suvmax: 5.1), paraaortic-aortocaval (suvmax: 8), common iliac (suvmax: 7.7), left external iliac (suvmax: 9), and left inguinale (suvmax: 7.15) lymph nodes.

**Figure 2:**
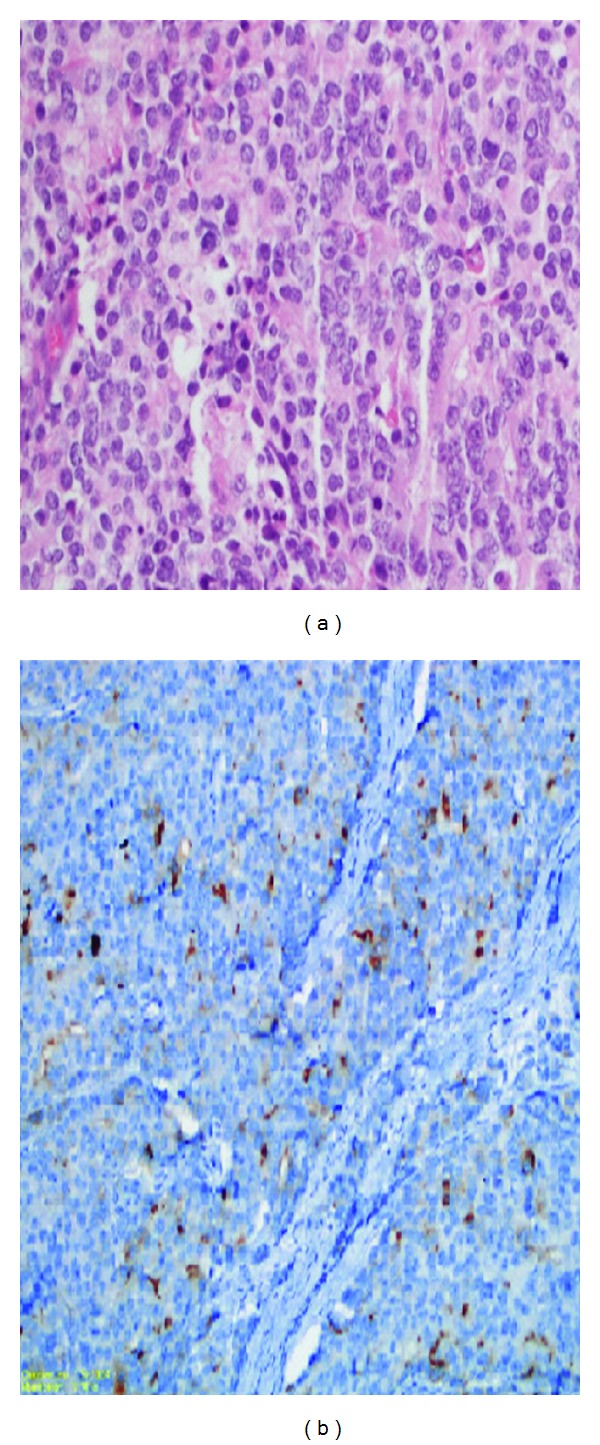
(a) Histopathological diagnose was acinar type adenocarcinoma, gleason grade was 4 + 5, perineural invasion (+) (hematoxylin-eosin, magnification X200). (b) The tumor cells stained strongly positive for prostate-specific antigen and stains for neuron-specific enolase, synaptophysin, chromogranin, *S*-100, pancytokeratin, leukocyte common antigen, and carcinoembryonic antigen were negative (immunohistochemical staining X100).

A 59-year-old man admitted to a hospital, with left inguinale swelling. He did not have any complaints. His past medical history was unremarkable. It was diagnosed as inguinal hernia and the patient went to surgical operation. After the surgeon, the swelling was compatible with inguinal hernia. Three weeks after the surgery, the swelling repeated. Thus computerized tomography of the abdomen was made. It showed paraaortic, aortocaval, bilaterale parailiac, and left inguinale lymph nodes of which the biggest was 13 × 26 mm. The liver and spleen were not palpable and there was no other adenopathy. Digital rectal examination was normal. The rest of the physical exam was unremarkable. Laboratory evaluation revealed mild normocytic normochromic anemia with hemoglobin of 12.3 g/dL (range, 13.2 to 17.1). Renal and hepatic functions were normal. Whereupon a biopsy of the left inguinale node was taken, it was compatible with high grade adenocarcinoma of undetermined origin, investigation of primarily pulmonary or urogenitale systems was suggested for origin. After it, PET CT showed pathological increased FDG involvement of the left supraclavicular (suvmax: 4.5), aorticopulmonary (suvmax: 3.6), paratracheal (suvmax: 4.1), left tracheobronchial (suvmax: 5), left hilar (suvmax: 4.95), superior phrenic (suvmax: 5.1), paraaortic aortocaval (suvmax: 8), common iliac (suvmax: 7.7), left external iliac (suvmax: 9) and left inguinale (suvmax: 7.15) lymph nodes (Figure 1). Then tumoral markers of the patient was studied. CEA, CA19-9, and CA15-3 were normal but AFP: 8.3 (0–8), total serum PSA: 119 ng/mL (0–3.9), free PSA: >20.4 ng/mL (0–0.4). This time, guided prostate core biopsy was made and 12 biopsy materials were obtained. All of them were involved with tumoral cells. Histopathological diagnosis was acinar type adenocarcinoma, gleason grade was 4 + 5, perineural invasion (+), and the tumor cells stained strongly positive for prostate specific antigen, confirming its prostatic origin. Stains for neuron-specific enolase, synaptophysin, chromogranin, *S*-100, pancytokeratin, leukocyte common antigen, and carcinoembryonic antigen were negative (Figure 2). 

Bilateral orchiectomy was made. A bone scan showed normal uptake of radioactivity in the pelvic bones, thoraco-lumbar spine, and the ribs. Finally we defined the patient as prostatic adenocarcinoma with generalized lymph node metastases and started bicalutamide 50 mg, once a day. Total and free PSA levels and computerized tomography (CT) of chest, abdomen and pelvis were planned as follow-up parameter with a three-month interval. Three months later, his generalized adenopathy had completely regressed and serum PSA had decreased to normal level (0–4 ng/mL). 

## 3. Discussion

Some carcinomas of the prostate are slowly growing and may persist for long periods without causing significant symptoms, whereas others behave aggressively. It is not known whether tumors can become more malignant with time. Both early and advanced carcinomas of the prostate are frequently asymptomatic at the time of diagnosis. Rarely patients present with signs of urinary retention (palpable bladder) or neurologic symptoms as a result of epidural metastases and cord compression. Lymph node metastases can lead to lower extremity lymphedema. As the axial skeleton is the most common site of metastases, patients may present with back pain or pathologic fractures. Our patient did not have any symptoms at initial presentation. 

Lymphatic metastasis of prostate occurs initially to the obturator nodes followed by perivesical, hypogastric, iliac, presacral, and para-aortic nodes. Lymphatic spread can best be assessed by surgical exploration; the frequency with which it occurs correlates with the size, grade, and the level of serum PSA. Only about one tenth of tumors with a grade of less than 5 have lymph node involvement, while more than 70 percent of tumors with Gleason grade 9 or 10 have coexisting lymphatic invasion at the time of diagnosis. Lymphatic metastases are present in fewer than 10 percent of patients in whom the serum PSA level is < 10 ng/mL [3]. Except for bone involvement, distant metastases are relatively rare at diagnosis and include supraclavicular, mediastinal, pulmonary, and retroperitoneal metastases, which seldom are the first evident clinical manifestation of prostate adenocarcinoma [4]. In the literature, we found few case reports. Metastasis to the cervical lymph nodes is seen in about 0.4% of patients with prostate cancer [5, 6]. In a large series of mediastinal metastasis, only 1% originated from the prostate [7]. However, our patient's PET CT scans showed widespread involvement of the lymph nodes by the tumoral cells. First of all, we thought lymphoma was suitable for our patient because superficial and widespread lymphadenopathy is a symptom of systemic disease such as infection, collagen disease, and lymphoproliferative disorders, including acute and chronic lymphocytic leukemia and malignant lymphoma [8]. But our patient did not have any infectious symptoms or B symptoms (fever, perspiration, and weight loss) for malignant lymphoma. The avidity of tumor cells for PSA on immunohistochemical staining initially raised suspicion for prostate cancer, which was later confirmed by biopsy of the prostate.

The prognosis and response to treatment of patients with lymph node involvement does not seem to differ from that of other patients with metastatic prostate cancer [9]. 

In summary, the diagnostic difficulty in the present case was a result of the fact that the patient presented supraclavicular, mediastinal, hilar, pulmonary, and retroperitoneal involvement as the initial manifestation of prostate cancer. However, the present case suggests that in any male older than 50 years of age with generalized lymphadenopathy, prostate carcinoma metastases should also be considered, especially if urologic symptoms and elevated PSA levels are also present. A suspicion of prostate cancer in male patients with adenocarcinoma of undetermined origin is important for an adequate diagnostic and therapeutic approach.
